# Optimizing Health Information Technologies for Symptom Management in Cancer Patients and Survivors: Usability Evaluation

**DOI:** 10.2196/18412

**Published:** 2020-09-21

**Authors:** Emily G Lattie, Michael Bass, Sofia F Garcia, Siobhan M Phillips, Patricia I Moreno, Ann Marie Flores, JD Smith, Denise Scholtens, Cynthia Barnard, Frank J Penedo, David Cella, Betina Yanez

**Affiliations:** 1 Department of Medical Social Sciences Northwestern University Chicago, IL United States; 2 Department of Preventive Medicine Northwestern University Chicago, IL United States; 3 Department of Physical Therapy and Human Movement Sciences Northwestern University Chicago, IL United States; 4 Department of Psychiatry and Behavioral Sciences Northwestern University Chicago, IL United States; 5 Department of Medicine Northwestern University Chicago, IL United States; 6 Departments of Psychology and Medicine University of Miami Coral Gables, FL United States

**Keywords:** cancer survivorship, eHealth, patient-reported outcomes, digital health, symptom management, supportive care

## Abstract

**Background:**

Unmanaged cancer symptoms and treatment-related side effects can compromise long-term clinical outcomes and health-related quality of life. Health information technologies such as web-based platforms offer the possibility to supplement existing care and optimize symptom management.

**Objective:**

This paper describes the development and usability of a web-based symptom management platform for cancer patients and survivors that will be implemented within a large health system.

**Methods:**

A web-based symptom management platform was designed and evaluated via one-on-one usability testing sessions. The System Usability Scale (SUS), After Scenario Questionnaire (ASQ), and qualitative analysis of semistructured interviews were used to assess program usability.

**Results:**

Ten cancer survivors and five cancer center staff members participated in usability testing sessions. The mean score on the SUS was 86.6 (SD 14.0), indicating above average usability. The mean score on the ASQ was 2.5 (SD 2.1), indicating relatively high satisfaction with the usability of the program. Qualitative analyses identified valued features of the program and recommendations for further improvements.

**Conclusions:**

Cancer survivors and oncology care providers reported high levels of acceptability and usability in the initial development of a web-based symptom management platform for cancer survivors. Future work will test the effectiveness of this web-based platform.

## Introduction

Improvements in early cancer detection and treatment effectiveness have led to an increasing number of individuals living with and surviving cancer in the United States. By 2029, it is estimated that the number of individuals affected by cancer in the United States will exceed 21 million [1]. Despite the advances in early detection and treatment success, the survival benefit can be offset by chronic and debilitating cancer-related side effects and symptoms. Unmanaged side effects and symptoms can compromise long-term treatment success, and can lead to worsening health-related quality of life and clinical outcomes among patients diagnosed with cancer [2,3].

Assessing patient-reported outcomes (PROs), or the patient-reported experience of symptoms and concerns, is key for comprehensive cancer care. PRO assessment to guide care can improve patient satisfaction and has been associated with enhanced clinical outcomes [2]. Integrating the systematic monitoring of PROs into care provided in clinical settings can improve symptom monitoring and enhance opportunities for patient engagement in symptom self-management. When symptoms detected by measuring PROs are managed quickly by the care team, adverse events and toxicities may be more effectively prevented, reversed, or managed [2,4].

Although the use of PROs to inform clinical care is not novel, newer technologies can facilitate the collection of PRO data, which can then be used more efficiently to improve patient care [5]. Recently, Basch and colleagues [6] implemented a web-based program that allowed patients to routinely and remotely report ePRO symptoms to their clinicians. Patients in the trial showed improved symptoms and longer survival within a randomized single cancer center trial, demonstrating the potential for web-based ePROs to have a favorable impact on survival. ePROs have also been embedded into clinical care through electronic health records (EHRs). For example, within the Northwestern Medicine health care system, patients in ambulatory oncology complete ePROs of physical symptoms and psychosocial concerns through a patient-facing web portal (Epic MyChart) that is linked to the EHR. PROs are incorporated into the EHR, and EHR-based alerts automatically trigger to inform clinicians of their patients’ clinically elevated ePRO symptoms [7-9]. Patients who triggered alerts based on their elevated ePRO scores had more health care service encounters in the ensuing month, demonstrating that ePROs may be an effective method of connecting patients with necessary care [7].

Collectively, the literature supports the feasibility and preliminary efficacy of systematic ePRO symptom monitoring and the integration of ePROs into EHRs. As part of the National Cancer Institute (NCI) Cancer Moonshot [10], our team is implementing and evaluating a fully EHR-integrated oncology ePRO symptom assessment and management program across Northwestern Medicine’s 7-hospital health care delivery system. The research team worked with patients diagnosed with cancer, and identified an opportunity to improve these patients’ access to relevant, concise, focused symptom management resources by creating an innovative electronic resource of patient treatment–related education and supportive care resources. Although there are numerous cancer online resources available, cancer patients have expressed a preference for obtaining health information from reputable websites such as those associated with universities and health systems [11]. Our web-based platform, called “My NM Care Corner,” moves beyond providing only symptom management information and further provides patients with tailored feedback on their recently completed PRO assessments that is linked to evidence-based symptom management information, including resources to enhance health-related quality of life. The My NM Care Corner platform will be available in both English and Spanish, and is integrated with the Epic EHR system; thus, there is high potential for dissemination across health systems using Epic.

The purpose of this research was to evaluate the usability of this new health information technology tool in preparation for its implementation. In this paper, we briefly describe the development of My NM Care Corner and present findings from the usability trial of the My NM Care Corner website.

## Methods

### Prototype Development

The prototype of My NM Care Corner was designed and developed by a multidisciplinary team with expertise in design, clinical science, and information technology. Because the ultimate goal of this research is for the web-based platform to become a part of standard care, we followed the style guide requirements mandated by the Northwestern Medicine health care system. The style guide requirement included guidelines on font size, colors, and website layout. The requirements for the clinical content provided by My NM Care Corner were primarily drawn from the NCI-funded “Improving the Management of symPtoms during And following Cancer Treatment” (IMPACT) consortium, which focused on providing patient-centered information on common cancer-related symptoms (eg, pain, fatigue, nausea, physical functioning, insomnia, anxiety, depression, constipation), evidence-based approaches to symptom management, and resources available to cancer patients and survivors at Northwestern Medicine.

The primary features of My NM Care Corner are described in Table 1. The dashboard, symptom library, and patient resources (Enhancing Well-Being, Diet & Nutrition, Support Services & Resources, Financial & Practical Matters, Treatment & Symptom Management) were included to provide health-related quality of life–enhancing materials and to provide educational materials that focus either on symptom management or health-related quality of life, which are our primary study outcomes for the larger trial. The welcome video was included based on user feedback gathered early in the course of this usability testing. The majority of the content in My NM Care Corner was derived from previous technology-assisted pilot studies that focused on improving patient symptom management and health-related quality of life among diverse patients with cancer diagnoses [12-16]. In My NM Care Corner, symptoms flagged as elevated based on ePRO responses are visible to users when they access their dashboard. Because patients may be interested in reading about symptoms that were not elevated on their most recent ePRO responses, information about all symptoms was placed under the “Symptoms” tab, and information about how to deal with symptoms and enhance well-being were placed under the “Patient Resources” tab. Users can also mark information as “favorite,” which will be saved under the “My Favorites” tab. The health care system–specified style guide and the requirements from the grant provided a framework for the prototype, and the user interface was examined for usability and perceived usefulness within this framework.

**Table 1:** Features of My NM Care Corner.

Section			Description^a^	
Welcome Video			Brief user engagement video that orients patients to My NM Care Corner	
Dashboard			Displays information about symptoms that are flagged based on ePRO^b^ completion	
Symptom Library			Includes information about the following symptoms: pain, fatigue, depression, anxiety, insomnia, physical function, nausea and vomiting, shortness of breath, constipation, diarrhea	
**Patient Resources**				
	Enhancing Well-Being		Includes information about talking with your health care team, social support, stress management, physical activity, problem solving, smoking cessation and substance abuse, sun protection, supportive oncology, managing hair loss, and body image concerns	
	Diet & Nutrition		Includes information about the importance of healthy eating, healthy foods, maintaining a balanced diet, managing weight loss, managing weight gain, dealing with loss of appetite, nutrition tips	
	Support Services & Resources		Includes information about supportive oncology at Northwestern Medicine, local support groups and communities, one-to-one support from cancer survivors, support online and by phone	
	Financial & Practical Matters		Includes information about managing the cost of treatment, managing legal and workplace issues, managing transportation during treatment	
	Treatment & Symptom Management		Includes information about palliative care, integrative care, cancer rehabilitation services, cancer survivorship services	
My Favorites			Allows the user to save pages from the Patient Resources section to view later	
Contact Us			Provides information on contacting the study staff by email or telephone	

^a^All content is available in English and Spanish and is audio-accessible.

^b^ePRO: electronic patient-reported outcomes.

### Recruitment

We recruited a convenience sample of participants in the fall of 2019. Inclusion criteria for cancer survivors required that individuals have a past or present cancer diagnosis, a history of receiving care at Northwestern Medicine’s cancer center, and the ability to speak and read English. Inclusion criteria for staff and clinicians were current employment at Northwestern Medicine, with at least a portion of their work being focused in the cancer center.

Although staff are not among the intended end users of My NM Care Corner, recruitment to behavioral clinical trials is often enhanced by clinician investment in interventions [17]. We also included staff in this early qualitative work to ensure that My NM Care Corner content would enhance and not contradict the care they provide patients. Staff and clinician participants were recruited via direct email invitations. We recruited 10 patient participants and 5 staff participants based on research demonstrating that 15 participants typically find 90%-97% of existing usability problems [18].

### Ethics Approval

The research procedures were initially reviewed by the university Institutional Review Board, and it was determined that the study was exempt from full review. The research procedures did not meet the definition of human subjects research because the study focused on participant reactions to the website rather than on the participants themselves. To ensure ethical conduct, participants were provided with an overview of the study and provided verbal consent prior to participation.

### Procedures

**Figure 1:**
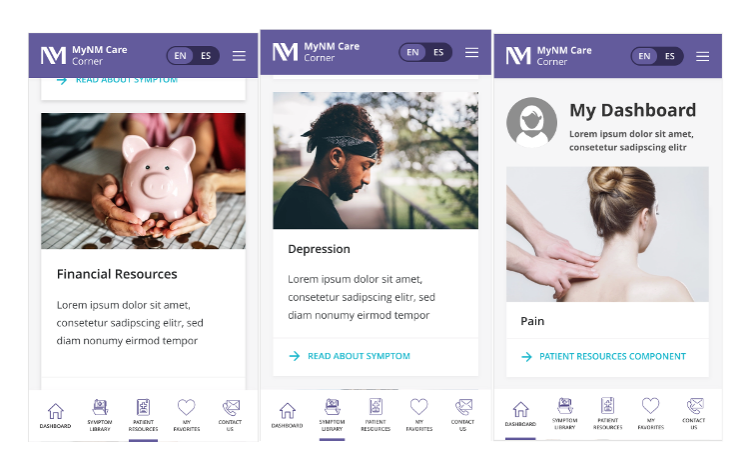
Screenshots of the mobile-friendly My NM Care Corner prototype.

Patient participants were asked if they typically look up health information on a smartphone or on a computer. They were then introduced to a prototype of My NM Care Corner that matched their preferred device (smartphone or computer), and were instructed to think aloud while completing specific tasks (see Figure 1 for representative screenshots of the smartphone prototype, and Multimedia Appendix 1 for screenshots of the computer prototype). Specific tasks included: (1) finding information about fatigue, (2) finding information on diet and nutrition, and (3) sending a message to the study staff. After completing these tasks, participants were shown the alternate version of the web-based platform (smartphone or computer) and were prompted to find the same information. Staff participants were first shown the computer-based prototype and were instructed to complete the same tasks as the patient participants. Staff participants were shown the smartphone prototype of the website, but because the intent of staff testing was more about content acceptability and organization rather than usability, they were not asked to find information on the smartphone prototype.

Following prototype testing, all participants participated in a brief (approximately 15 minutes) interview that addressed participant impressions of My NM Care Corner, including ease of use and perceived usefulness, and prompted participants to generate ideas about ways to improve the design of the web-based platform. Following the brief interview of the user’s impression of My NM Care Corner, participants completed usability questionnaires.

### Measures

The System Usability Scale (SUS) [19] and the After Scenario Questionnaire (ASQ) [20] were administered to provide quantitative measurements of usability.

The SUS is a 10-item questionnaire that prompts participants to rate their level of agreement with statements related to system usability. Example items include “I thought the system was easy to use” and “I found the system unnecessarily complex.” Participants respond to items on a 5-point scale ranging from “strongly agree” to “strongly disagree,” and after scoring procedures, the total scores on the scale range from 0 to 100. Past research has demonstrated that a SUS score above 68 is indicative of above average usability [21].

The ASQ is a 3-item questionnaire that prompts participants to rate their satisfaction with the usability of a program. Example items include “Overall, I am satisfied with the amount of time it took to complete the tasks in this scenario” and “Overall, I am satisfied with the ease of completing the tasks in this scenario.” Participants respond to items on a 7-point scale ranging from 1, which indicates “strongly disagree,” to 7, which indicates “strongly agree.” After scoring, the total scores range from 1 to 7, with higher scores indicating higher levels of satisfaction with usability.

### Analytic Plan

This study included both qualitative and quantitative data that we analyzed using a mixed methods approach. We adopted this approach to usability testing because, while quantitative data can identify usability issues and dissatisfaction with program design, qualitative data provide information about the root of the usability issues and potential methods for program optimization. The usability testing session interviews served as the primary source of qualitative data. Interviews were transcribed, and the transcripts were analyzed using a conceptual content analytic approach. Coding of these transcripts was completed using NVivo [22]. Quantitative data collected included a demographic questionnaire, rates of task errors, rates of repeat task errors, and scores on SUS and ASQ. These data were analyzed using descriptive statistics.

## Results

### Participants

Ten patients and five clinical staff participated in the study. In regard to sex, 5 patients identified as cisgender males and 5 identified as cisgender females. The mean age was 55.3 years (SD 12.7) ranging from 39 to 74 years. The majority of patients identified as non-Hispanic White (n=8), with one patient identifying as Hispanic White and one identifying as Hispanic but declining to identify their race. Four of the patient participants were breast cancer survivors, two were renal cell carcinoma survivors, and the other four patient participants reported diagnoses of melanoma, ovarian cancer, tonsil cancer, and base of the tongue cancer, respectively. Three of the 10 patient participants were in active treatment (which included adjuvant chemotherapy), and the mean time since diagnosis was 36.8 months, ranging from 8 to 83 months.

Staff participants included two clinical psychologists, one social worker, one patient educator, and one medical oncologist. One of the five staff participants was a cancer survivor and one of the five staff participants identified as cisgender male (the remaining four identified as cisgender female). Four of the five staff participants identified as non-Hispanic White and the remaining staff participant identified as Asian. The mean staff participant age was 44.4 years (SD 11.7), ranging from 30 to 61 years.

### Usability Testing Performance

Nearly all participants (9 out of 10 patient participants and 4 out of 5 staff participants) were able to complete all tasks on My NM Care Corner as assessed on the usability protocol. In the first task of the protocol, participants were prompted to find information about fatigue and all participants were able to find this symptom information without difficulty. However, 7 of the 10 patient participants and all 5 of the staff participants experienced some difficulty completing the second task of the protocol, in which they were asked to find information on diet and nutrition; most of these participants first looked under the Symptom section of the website before exploring the Patient Resources section. Despite the high level of initial confusion, only 2 of these participants (1 patient participant and 1 staff participant) did not persist through searching for the information and requested guidance from the interviewer to resolve. The participants who experienced these errors reported that they would have likely been able to find the requested information if they had been provided with an orientation or introduction to My NM Care Corner. All participants were quickly able to complete the third task, in which they were asked to find where they could send a message to the study team.

Completion times for the usability tasks were highly variable. The variability in completion time appeared to be primarily related to individual styles in reviewing the presented information (eg, some participants skimmed through text, while others read all of the content presented) and did not appear to be a reliable metric for program usability. Although the participants had been instructed to complete the tasks and comment primarily on the navigation and general design of My NM Care Corner, some participants read all of the content closely and provided detailed feedback on the content.

There were very low rates of repeat errors. Although the majority of participants had some level of difficulty finding information about diet and nutrition on their first pass through My NM Care Corner, none of the participants had difficulty finding this information in their second pass through the web-based platform, indicating a high level of learnability.

### Usability Feedback

Quantitative usability feedback was generally positive, with a mean score on the SUS of 86.6 (SD 14.0, range 52.5-100), indicating above average usability. The mean score on the ASQ was 2.5 (SD 2.1, range 1-5.6), indicating a relatively high level of satisfaction with the usability of the My NM Care Corner prototype.

Qualitative analyses revealed several strengths noted in the initial design of My NM Care Corner, and there was an overwhelming positive response to the functions of the web-based platform. Primarily, participants appreciated the desktop and mobile-friendly format, information on self-management strategies, and noted that the Northwestern branding enhanced trust in the information. Participants voiced appreciation for having quick access to personally relevant symptom information that is available 24 hours a day.

Most participants liked the concept of the dashboard, which served as the landing page. All of the patient participants were familiar with completing PRO measures through the EHR, and many wondered where that information was transmitted. Several participants reported that being able to access personally relevant symptom information would be beneficial to them. One patient (Pt 104) noted, “I like the idea of the dashboard a lot. I mean it’s nice to know that when I provide information it’s not just going onto some file somewhere and no one’s really reading it.”

Anticipated uses of My NM Care Corner by patients included being able to mark as favorite and review information about issues of interest. Respondents also highlighted the benefit of knowing a trusted source of information. They voiced interest in being able to get information about both symptoms and self-management strategies to try between appointments. As Pt 108 commented:

I think “Strategies to try at home” should be really big because I’m looking on a computer about fatigue because I’m not in front of my doctor and I can’t say, “Hey, what can I do about fatigue?” And strategies at home are what I think exactly what I think I’d be looking for.

Patient participants also envisioned themselves using My NM Care Corner to ease anxiety by reviewing information about common symptoms, especially during “off hours” of the clinic. They anticipated that some patients would use the site to orient themselves when coping with a new diagnosis.

Staff participants were also generally enthusiastic about the features of the web-based platform and viewed it as a valuable supplement to care. Staff participants envisioned using My NM Care Corner as a referral resource for patients and to reinforce concepts discussed during appointments. Additionally, there was recognition that physicians do not always discuss self-management strategies at length with patients before prescribing medications to help manage side effects of treatment, and that this program could help to reinforce self-management. One staff participant (Pt 204) noted:

I think if we had something that was NM [Northwestern Medicine] branded, looks like everything else that gets sent to them. It would be much easier to say “Stay off the internet, get off breast cancer chat groups. Go here for a start.”

Similarly, another staff participant (Pt 205) commented:

I think it rounds out the care. I think it allows patients to look at a list of potential options that could be utilized for constipation and decide what they think resonates with them. Instead of like, “My doctor told me that I should do this, so can I do this also? Or is that not something I should do?” I think it allows a little bit more of a portfolio.

Participants voiced appreciation for the web-based platform being available in both a desktop and mobile-friendly format. Pt 104 noted, “But it’s nice to know that it doesn’t really vary because sometimes you have a desktop version and it’s nothing like the mobile version and you can’t get through anything on the mobile version.” There were no notable differences in feedback from patient participants who viewed the mobile version of My NM Care Corner first relative to those who viewed the desktop version of My NM Care Corner first. Feedback on both modes of delivery was generally positive. This appeared to be more related to how participants typically accessed health information rather than differences in formatting between the computer and mobile-friendly versions.

There was also positive reception of the general design, and many found that they had the intuitive ability to navigate through My NM Care Corner. As one participant (Pt 107) commented, “Well it’s not, it’s not hard to navigate at this point, per se. At this point I mean, it’s pretty logical.” Similarly, another participant (Pt 104) stated, “It was pretty self-explanatory. Everything is listed at the bottom so it’s easy to find.”

The format was perceived as easy to follow for potential users who may be feeling fatigued or unwell. As a staff participant (Pt 201) noted:

I like the way that, the boxes kind of give you a little bit of information of what the next page will include. I think that’s really helpful because if you’re especially not feeling well, it’s easy to get overwhelmed or distracted so I like the simplicity of it, it’s not going into a lot of dialogue where you have to kind of search for what it is that you need.

Avoiding information overload is key when designing health information programs, and most participants found this program usable in that regard.

A majority of participants described the design of My NM Care Corner as “neat” and “clean looking.” A few participants indicated that the design of the web-based platform felt unfamiliar to them. Although comments such as the following from Pt 106, “It was very confusing to look to see, you know, if you don’t find it here you could find it you know, because usually what I have experienced with websites is that you have one area where … you have everything,” were relatively infrequent, this sentiment points to the need for clear guidance or training on how to use My NM Care Corner and for what it can be used. Indeed, the most commonly identified areas for improvement were increasing the font size, adding a search tool, and adding instructions or an orientation on what types of resources and information one can expect to find in this web-based platform.

As noted in the Methods section, health system style guides and health system branding were used on My NM Care Corner. This branding appeared to enhance participants’ trust of the information presented. As Pt 108 commented:

Nutrition, fatigue, those are things cancer patients are going to be looking for. People are going to be looking for when they get on. It’s branded with Northwestern Memorial so I’m like “Oh okay, I know I’m in the right spot.”

However, some participants felt that the standard format of the web-based platform was too clinical and not emotionally engaging. As Pt 101 noted, “I don’t know, something that makes me want to not be so, I guess, clinical. Um, make it a little more happy.”

## Discussion

### Principal Findings

Results of this study demonstrate that the My NM Care Corner prototype was usable and engaging for both cancer patients and cancer center staff. Both patients and staff reported a high level of perceived utility of My NM Care Corner, and expressed ways that the platform could be useful to a variety of patients receiving care within the cancer center.

This study presented an opportunity to examine how incorporating hospital-required branding into formative design work informed the development of the prototype. While My NM Care Corner is being developed for a clinical trial, our team explicitly aimed to develop the platform in a manner that could result in it becoming standard of care following completion of the trial. Thus, we designed the platform to meet the needs of patient stakeholders, provider stakeholders, and health system stakeholders. Designing to the requirements of these different stakeholder groups meant that competing needs had to be prioritized and weighed against one another to make design decisions [23]. For example, although some participants found the color schemes less pleasing than desired, the branding of the web-based platform as part of the health system took priority. Consistent with past research on perceptions of health care brand images [24,25], many participants reported trust in the prototype due to its Northwestern Medicine branding.

Because My NM Care Corner is being designed for all adult patients at our cancer center, there were initial discussions about the level of detail to be presented (eg, related to specific cancer types, treatments, or phases of the care continuum). There are challenges inherent in designing programs that feature universally accessible informational resources [26]. It is well established that patients’ needs change throughout their journey with a cancer diagnosis [27]. Attempts to provide patients with information to meet all possible needs can quickly leave patients feeling overwhelmed, resulting in informational resources being underutilized [28,29]. The resulting model of presenting core symptoms and side effects (eg, pain, fatigue, nausea, physical functioning, insomnia, anxiety, depression, constipation) was deemed useful by participants, who primarily found the specificity of information to be appropriate.

Feedback gathered during sessions prompted changes to interface design such as adding images to the landing page, changing the font sizes, and designing a more recognizable hierarchy of headings and subheadings based on font. These changes were integrated into the current version of My NM Care Corner. Based on participant feedback, we also will include an introductory video to the website to enhance user engagement. Additional participant feedback such as a request to add a search function to the website will be added to future versions.

The cancer patient participants in the study were particularly interested in receiving tailored information based on their ePRO scores, and some participants commented that they had wondered why they had not previously received this information. This interest highlighted the commonly held desire of patients to receive personalized feedback and instruction on self-management practices through web-based and eHealth platforms [30-32]. My NM Care Corner was generally perceived as filling a gap in the support they received through the provision of tailored information.

### Limitations

Participants were recruited based on convenience sampling and all participants spoke English as their primary language. We plan to release My NM Care Corner in both English and Spanish, and although the navigation through the site is unlikely to be significantly impacted by language, there could be cultural preferences that were not identified in this round of testing on an English-language prototype [13,33]. The participants were not homogenous in regard to gender, cancer type, or age; however, the majority of participants identified as non-Hispanic White. In this study, perceived utility of the platform did not appear to vary by cancer type, but because this was a small sample, we cannot definitively conclude this point. We will examine perceived utility and use of the platform by cancer type in future research. Additionally, although the purpose of this phase of usability testing was on navigation through the web-based platform, some participants expressed interest in reading all available written content to rate the usefulness and applicability of the content. Because content was not fully developed at the time of the usability testing sessions, we were unable to subject the entire content to usability testing. Thus, although we determined that the prototype of My NM Care Corner was rated as highly usable in concept and was viewed as generally easy to navigate, this phase of the usability testing did not extend to an examination of the usability of clinical content. However, the majority of content in My NM Care Corner was derived from previous technology-assisted pilot studies that focused on improving patient symptom management and health-related quality of life among diverse patients with cancer diagnoses, and thus have support for acceptability [12-16].

### Future Directions

The next step of this initiative is to launch the My NM Care Corner web-based platform to patients being served at our cancer center. We will evaluate the platform using a pragmatic type I effectiveness-implementation hybrid trial [34]. In this trial, we will compare patients who are randomized to receive access to My NM Care Corner to patients who are randomized to standard of care on symptom burden and health-related quality of life. We will also examine differences in health care use (eg, number of emergency department visits, hospitalizations, supportive care visits) and cancer care delivery (eg, unscheduled treatment breaks, provider response time to EHR-generated alerts), adjusting for disease-specific and demographic factors (eg, cancer type, level of education, age, and race). In the upcoming trial, patients randomized to receive standard of care will still complete their ePROs and will have clinical alerts sent to oncology but will not have access to the My NM Care Corner platform.

### Conclusion

This study demonstrates the development and positive evaluation of a prototype version of a web-based platform called My NM Care Corner that links web-based, EHR-integrated PROs with tailored, evidence-based symptom management information for patients diagnosed with cancer. The study also confirms interest in and perceived usefulness of this platform from both cancer patients and cancer center staff. Ongoing research is examining the uptake and effectiveness of the My NM Care Corner platform through a randomized trial.

## Appendix

Multimedia Appendix 1 Computer screenshots of My NM Care Corner.

## Abbreviations

ASQ: After Scenario Questionnaire
EHR: electronic health record
NCI: National Cancer Institute
PRO: patient-reported outcome
SUS: System Usability Score
