# Supplementary figures and images for: Optimizing Health Information Technologies for Symptom Management in Cancer Patients and Survivors: Usability Evaluation
Source: JMIR Form Res. 2020 Sep 21;4(9):e18412. doi: 10.2196/18412 (PMC7536600; doi:10.2196/18412)

Supplementary Materials #1

Computer screenshots of My NM Care Corner.
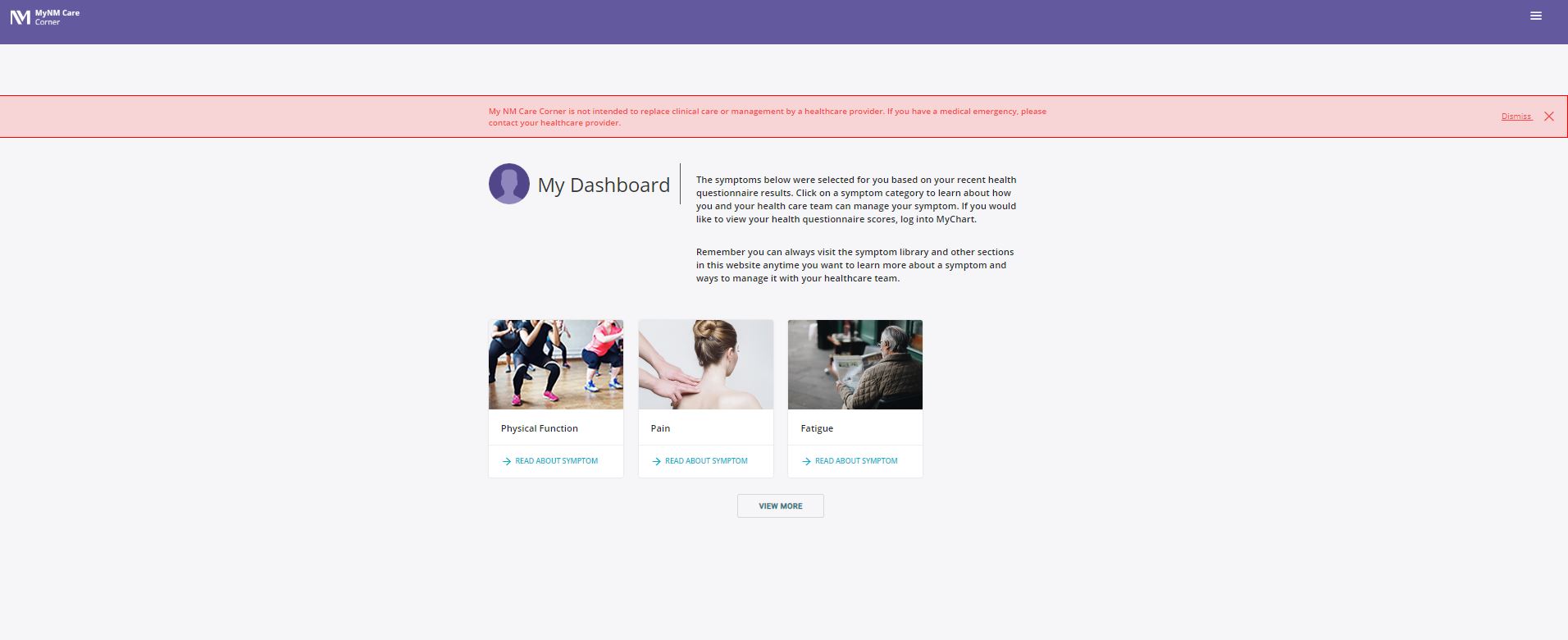


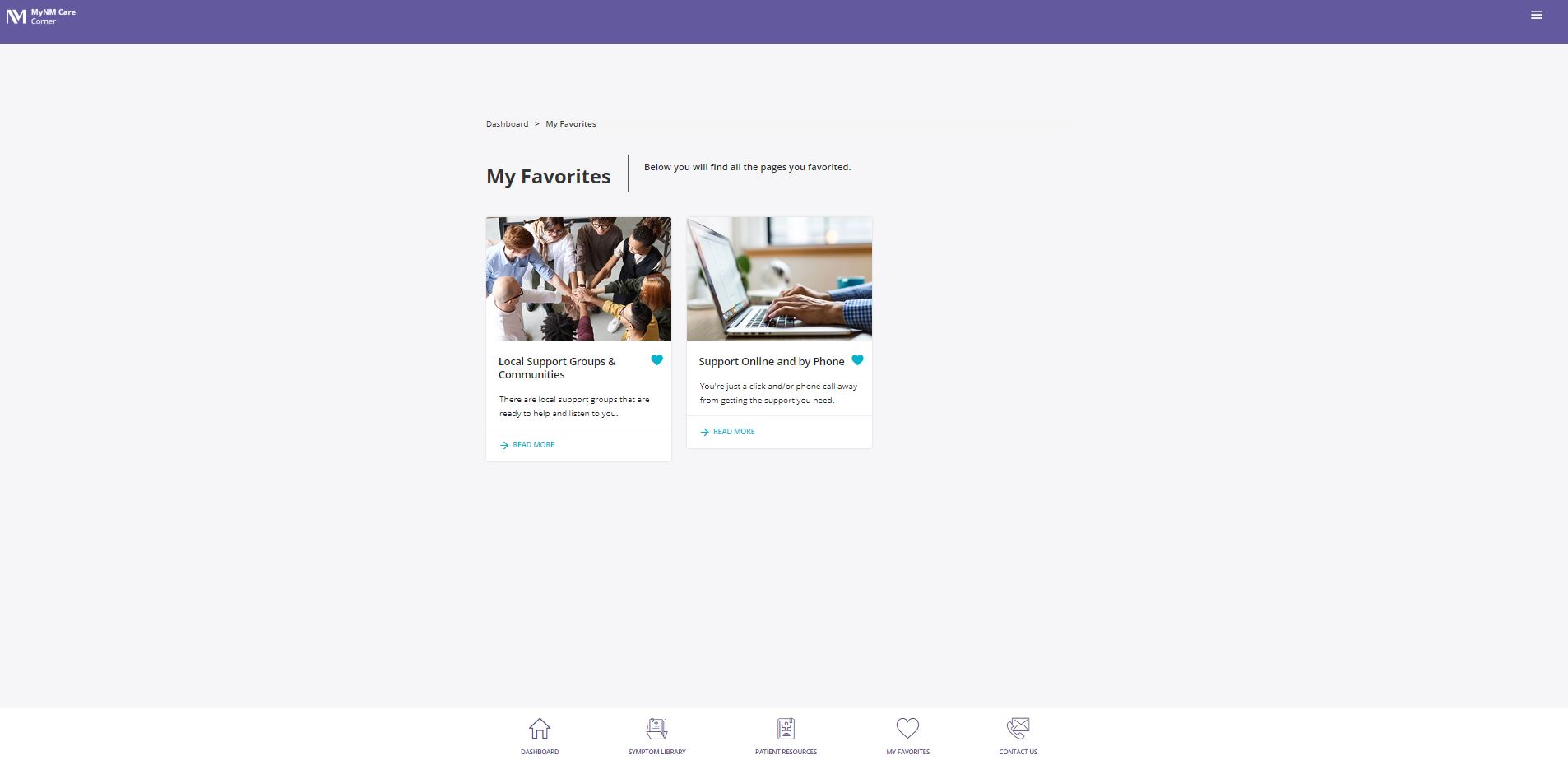

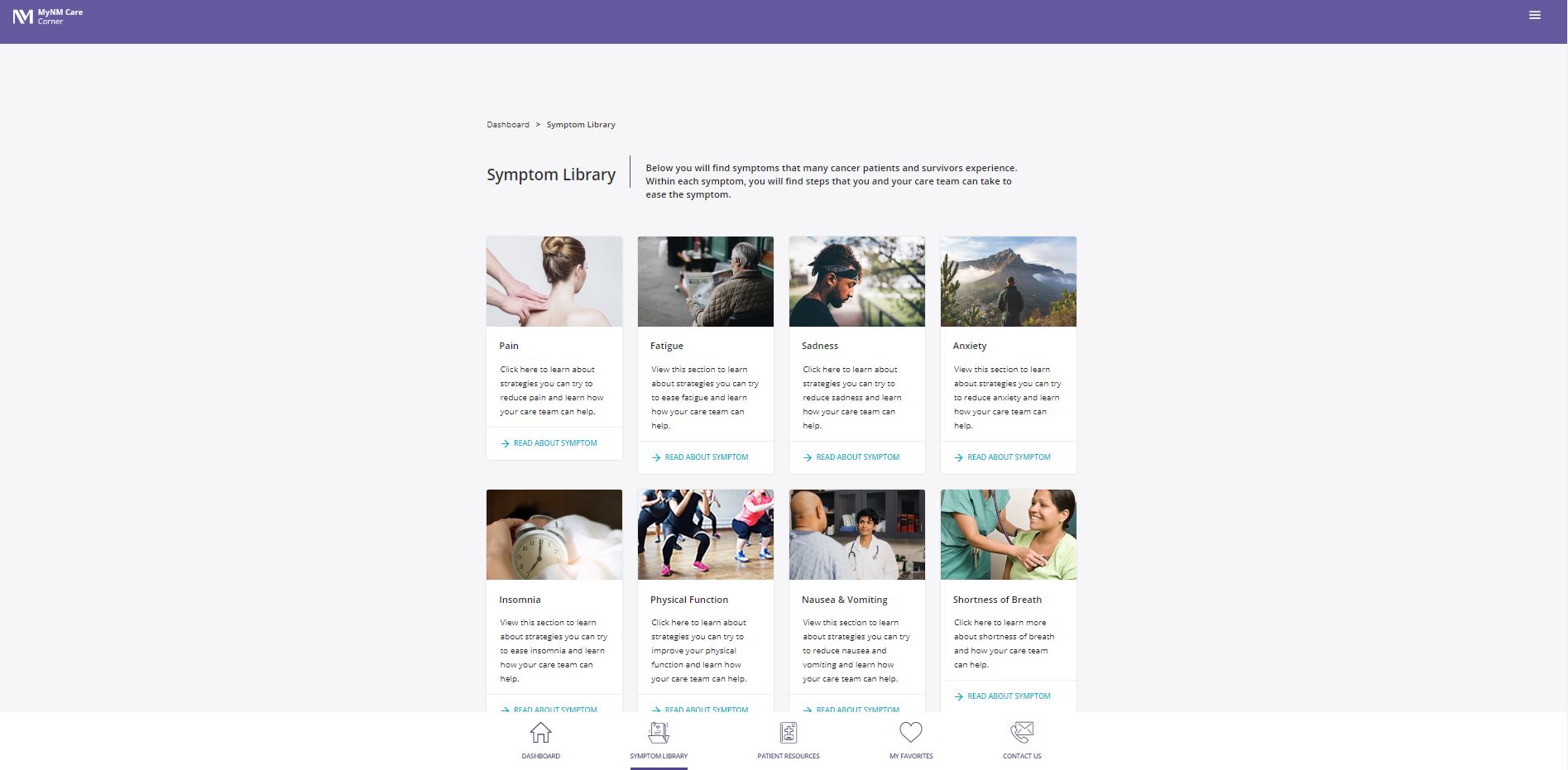

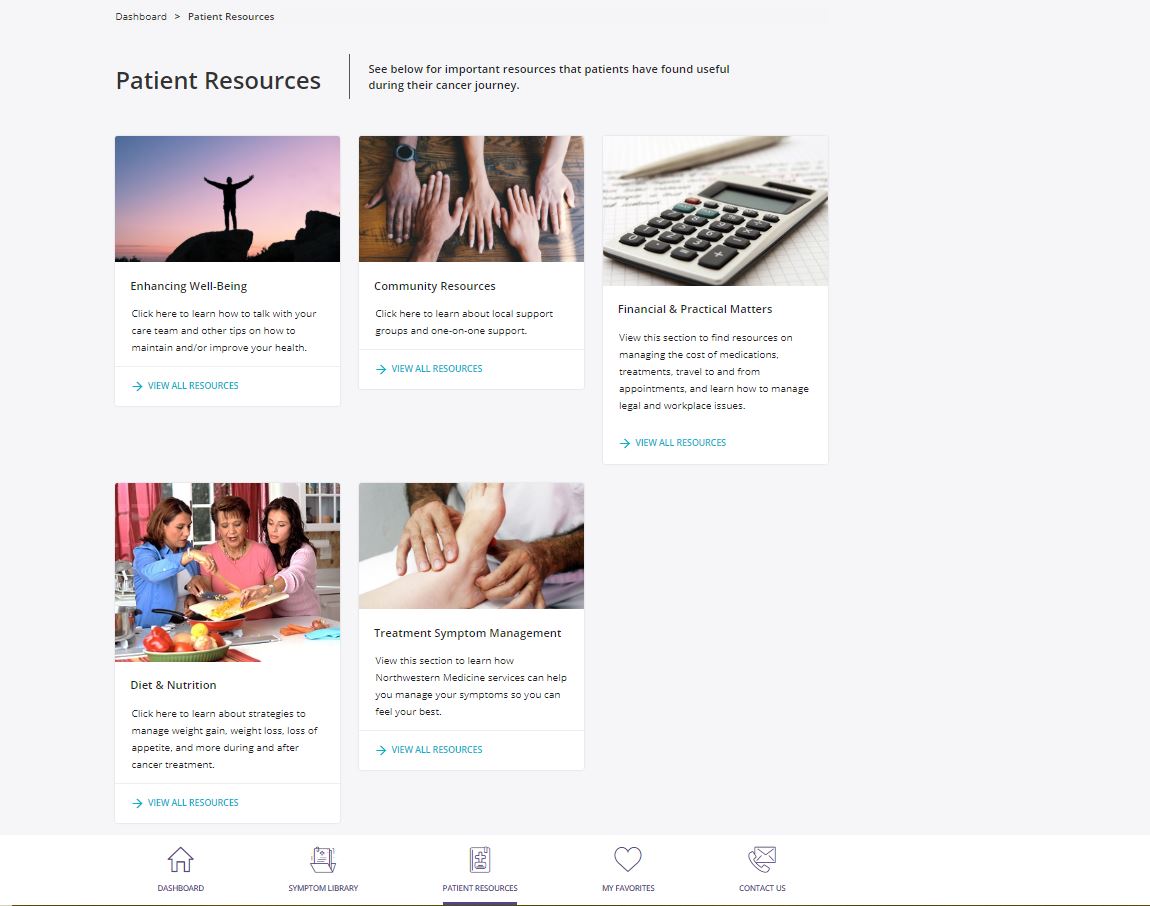
\


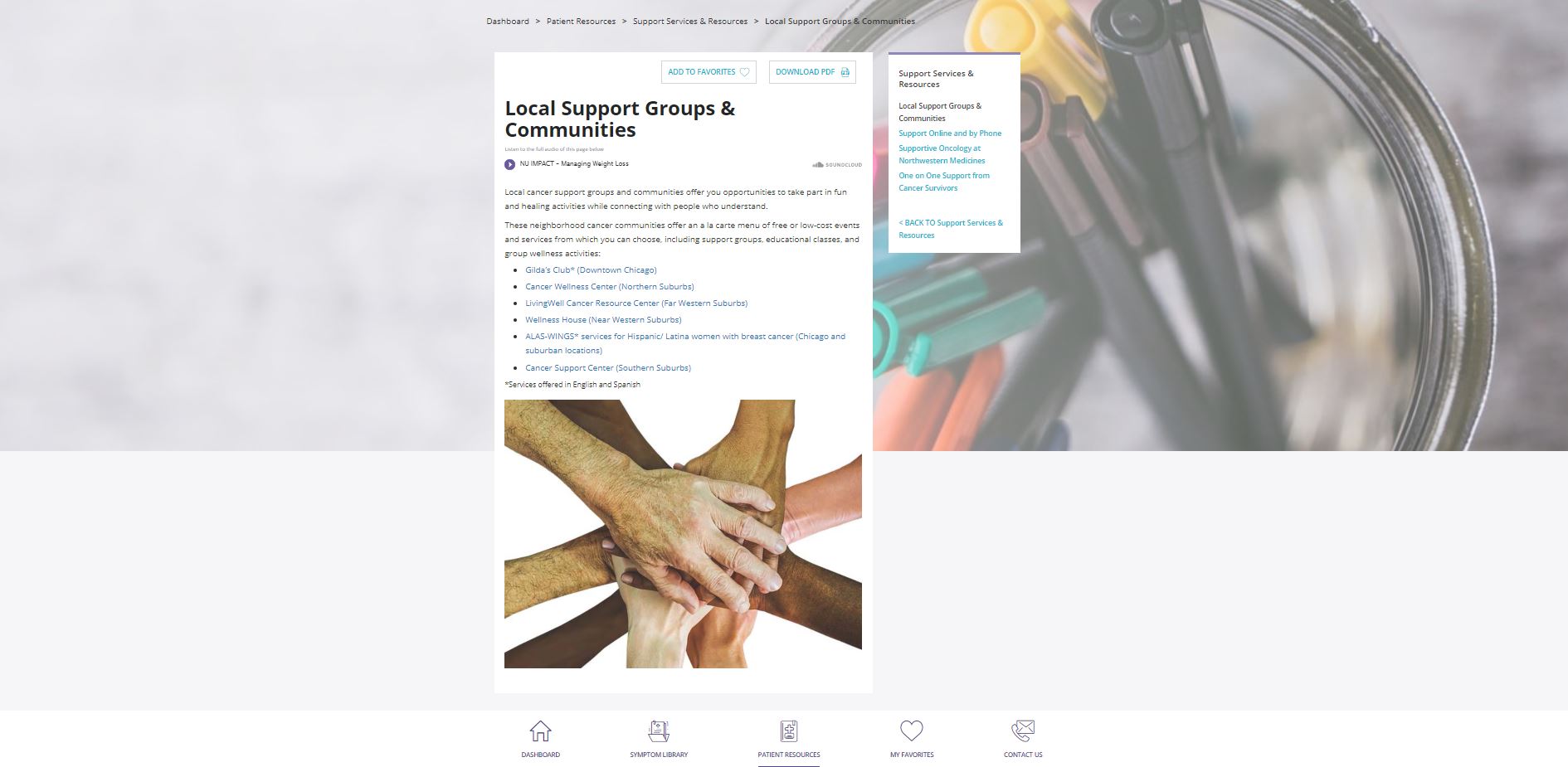

Supplement: Multimedia Appendix 1 [file formative_v4i9e18412_app1.docx]
